# Renal Transplant Patients Biopsied for Cause and Tested for C4d, DSA, and IgG Subclasses and C1q: Which Humoral Markers Improve Diagnosis and Outcomes?

**DOI:** 10.1155/2017/1652931

**Published:** 2017-01-15

**Authors:** James C. Cicciarelli, Nathan A. Lemp, Youngil Chang, Michael Koss, Katrin Hacke, Noriyuki Kasahara, Kevin M. Burns, David I. Min, Robert Naraghi, Tariq Shah

**Affiliations:** ^1^VRL Eurofins, Los Angeles, CA, USA; ^2^MNIT Foundation, Los Angeles, CA, USA; ^3^USC Keck School of Medicine, Los Angeles, CA, USA; ^4^Western University of Health Sciences, Pomona, CA, USA; ^5^University of Miami, Miami, FL, USA; ^6^BloodSource, Mather, CA, USA; ^7^UC Davis School of Medicine, Sacramento, CA, USA; ^8^CNSU College of Medicine, Elk Grove, CA, USA; ^9^St. Vincent Medical Center, Los Angeles, CA, USA; ^10^Transplant Research Institute, Los Angeles, CA, USA

## Abstract

The association between donor specific antibodies (DSA) and renal transplant rejection has been generally established, but there are cases when a DSA is present without rejection. We examined 73 renal transplant recipients biopsied for transplant dysfunction with DSA test results available: 23 patients diffusely positive for C4d (C4d+), 25 patients focally positive for C4d, and 25 patients negative for C4d (C4d−). We performed C1q and IgG subclass testing in our DSA+ and C4d+ patient group. Graft outcomes were determined for the C4d+ group. All 23 C4d+ patients had IgG DSA with an average of 12,500 MFI (cumulative DSA MFI). The C4d− patients had average DSA less than 500 MFI. Among the patients with C4d+ biopsies, 100% had IgG DSA, 70% had C1q+ DSA, and 83% had complement fixing IgG subclass antibodies. Interestingly, IgG4 was seen in 10 of the 23 recipients' sera, but always along with complement fixing IgG1, and we have previously seen excellent function in patients when IgG4 DSA exists alone. Cumulative DSA above 10,000 MFI were associated with C4d deposition and complement fixation. There was no significant correlation between graft loss and C1q positivity, and IgG subclass analysis seemed to be a better correlate for complement fixing antibodies in the C4d+ patient group.

## 1. Introduction

In* Humoral Theory of Transplantation* [1] Terasaki argued against Sir Peter Medawar's evidence for cellular rejection through thymus directed T-cell immunity that had for decades biased the transplantation community against antibodies as a cause of transplant rejection and loss. Terasaki first proposed a compelling hypothesis that linked antibodies (particularly to human leukocyte antigens (HLA)) with occurrence of transplant rejection. Antibody rejection was particularly associated with complement activation and shown specifically by the deposition of C4d on the kidney peritubular capillaries [2–4].

Interestingly, Terasaki showed in his studies a significant correlation of non-donor specific antibodies, HLA antibodies with poor outcomes [5–7], and later revealed the specific correlation of HLA donor specific antibodies (DSA) resulting in poor outcomes, that is, a more rigorous proof of the antibodies' role in rejection.

During the early days circa 2000, the elution of antibodies from rejected kidneys, biopsies, and C4d deposition results showed that both Sir Peter Medawar and Terasaki were correct. In several publications until the 1990s ([8] histological review) allograft dysfunction was accounted for by acute cellular rejection (ACR), and antibodies had a minor role with the exception of hyperacute rejection [9, 10]. Antibody mediated rejection (AMR) assumed a prominent role in allograft dysfunction and loss with the discovery of the complement protein C4d on the peritubular capillaries [2–4] and the principles described in* Humoral Theory of Transplantation* [1]. In fact, the association of antibodies was clearly shown by histologic and antibody examination of 232 transplant recipients, 67 undergoing acute dysfunction. In this study, 30% of the patients showed AMR only, 45% exhibited AMR plus cell mediated rejection (CMR), 15% CMR only, and only 10% acute tubular necrosis [11]. Clearly this data shows 75% of the patients had AMR. It is notable that antibody class switch from IgM to IgG is under the modulation of T-helper cells. Therefore, one can conclude that the T-cells are indirectly identified with AMR, and, of course, 60% of the group studied also had diagnosed CMR. Since AMR has been shown to be the prevalent component in graft rejection and loss, immunosuppressant drugs for AMR have become one of the most unmet needs for treatment. Graft rejection is currently controlled primarily by increasing T-cell immunosuppression, which one could argue is a good AMR immunosuppressant because of T-helper cell function in antibody formation. Albeit Rituximab, IVIg, Atgam, and Bortezomib seem to have an effect on B-cells and/or antibodies, there is no good plasma cell-targeting immunosuppressant agent.

With the discussion above as background, we have chosen to study antibody mediated rejection in a patient population that had allograft dysfunction with primary focus on C4d positive/DSA positive (C4d+ DSA+) patients. Our patient groups were long term graft survivors and had an average of >7 years after transplant at the time of dysfunction, biopsy, and DSA analysis. We examined 73 transplant recipients biopsied for transplant dysfunction, whereof 23 of these patients were diffusely positive for C4d (C4d+), 25 patients were focally positive for C4d, and 25 patients tested negative for C4d (C4d−). DSA test results for these patients were available within 1–10 days of the biopsy. In order to compare DSA and C4d results, we performed C1q and IgG subclass testing in our DSA+ and C4d+ patient group. Graft outcomes were determined for the C4d+ group. The antibody strength was ascertained by measurement of the mean fluorescence intensity (MFI) in the various tests.

Although there are commercially available kits for identifying C1q-binding HLA antibodies, IgG subclasses of HLA antibodies were measured by using several murine antibody clones recognizing human IgG subclasses. These clones have been tested by several other investigators with variable outcomes and correlations to the different subclasses [12–15]. In our hands, these clones behave differentially depending on the dilution tested and whether they were deployed in a direct or sandwich assay. Cross-reactivity was a major issue for us, whereas in other articles either cross-reactivity of clones was not determined [12, 13] or clones were described to be specific with minimal cross-reactivity [14, 15]. We primarily assessed the IgG subclasses using direct labeling. However, data is presented that suggests indirect “sandwich” assays could give a more specific result.

## 2. Material and Methods

### 2.1. Study Patients

Seventy-three renal transplant recipients (Table 1) were biopsied for cause and analyzed by immunofluorescence for C4d deposition in the peritubular capillaries (PTC) from frozen material. Twenty-three patients were diffusely positive (>50% PTC staining), 25 focally positive (20–50% PTC staining), and 25 negative (<20% PTC staining). The patients were chosen sequentially and reviewed retrospectively, with the requirement that a DSA test had been performed within ten days of the biopsy. All data review and study testing were performed under IRB approval.

### 2.2. C4d Immunostaining

Biopsied renal tissue was placed in Zeus fixative (Zeus Scientific, Branchburg, NJ), sectioned using a cryostat, and then stained as follows in a multistep procedure. Initially, the Zeus-fixed tissue was placed in Zeus fixative wash solution (Zeus Scientific) and sectioned in a cryostat at 4 *μ*m thickness, and the resultant sections were placed on poly-L-lysine-coated slides at room temperature, allowed to dry, and then fixed in cold acetone for 10 minutes. The tissue sections were then washed in PBS times 3 for 10 minutes each. The sections were then stained using a 3-step procedure as follows: (1) mouse anti-human C4d (Quidel Corp., San Diego, CA) was diluted 1 : 200, for 30 minutes and then washed in PBS; (2) FITC-conjugated rabbit anti-mouse immunoglobulin (Dako Corp, Carpinteria, CA) diluted 1 : 30, for 20 minutes, with interval wash in PBS; and (3) finally FITC-conjugated swine anti-rabbit immunoglobulin (Dako Corp), diluted 1 : 30, for 20 minutes, with final wash in PBS. Slides were then mounted with a cover slip in glycerol/Optimax solution and reviewed in an Olympus BX Fluorescence Microscope.

### 2.3. HLA Typing

HLA Class I & II antigens were detected using the standard monoclonal trays using the microcytotoxicity method (One Lambda, Inc., Canoga Park, CA). In the case of ambiguous results or uncertainty, additional testing using PCR-SSP was performed (One Lambda, Inc.) and (QIAGEN Inc., Valencia, CA).

### 2.4. HLA Single Antigen Bead (SAB) Specificity Analysis

All patients were tested for the presence of antibodies of IgG isotype using SAB Luminex technology (LABScreen Single Antigen, One Lambda, Inc.). The tests were performed according to the vendor's instructions using DTT-treated sera. Antibody specificity was analyzed manually using baseline mean fluorescent intensity (MFI) values. Positive MFI thresholds were defined on the basis of >500 MFI when a DSA was noted for a donor mismatched HLA antigen.

### 2.5. IgG Subclass Determination

SAB Luminex technology was used to determine the specificity of HLA Class I & II IgG antibody subclasses. The “direct” subclass assay was performed essentially in the manner of the standard SAB assay, with the replacement of the PE-labeled polyclonal goat anti-human IgG antibody with a PE-labeled monoclonal murine anti-human IgG subclass-specific antibody. DTT-treated graft recipient serum was reacted with SAB for 30 min, washed four times with 1x wash buffer, incubated with PE-labeled subclass-specific monoclonal antibody for 30 min, and washed twice before acquisition. The “sandwich assay” was performed with an unlabeled subclass-specific antibody and a PE-labeled secondary antibody. DTT-treated graft recipient serum was reacted with SAB for 30 min, washed four times with 1x wash buffer, incubated with murine antihuman IgG subclass-specific monoclonal antibodies for 30 min, washed four times, incubated with a polyclonal anti-murine IgG-PE conjugate for 30 min, and washed twice before acquisition. The antibodies used for the direct assay were purchased from Southern Biotech (Birmingham, AL) and are detailed in Table 2. The antibodies used for the sandwich assay were unlabeled murine monoclonal antibodies specific for human G1 (Clone HP6001, Millipore, Billerica, MA), G2 (Clone HP6002, Millipore), G3 (Clone HP6047, Alpha Diagnostic Intl. Inc., San Antonio, TX), and G4 (Clone HP6023, Millipore) subclasses and a goat polyclonal F(ab)2 anti-murine IgG-PE conjugate (R & D Systems, Minneapolis, MN). Positive control beads were produced by Acuimmune (Chatsworth, CA) using IgG1–IgG4 and IgM purified from myeloma plasma (Sigma-Aldrich, Saint Louis, MO) and coupled to Microplex Microspheres (Luminex, Austin, TX). Antibody specificity was analyzed manually using baseline mean fluorescent intensity (MFI) values. Positive MFI thresholds were defined on the basis of >500 MFI when a DSA was noted for a donor mismatched HLA antigen.

### 2.6. C1q SAB Specificity Analysis

C1q testing was performed using the commercially available kit (C1qScreen, One Lambda, Inc.) according to the manufacturer's instructions. Antibody specificity was analyzed manually using baseline mean fluorescent intensity (MFI) values. Positive MFI thresholds were defined on the basis of >500 MFI when a DSA was noted for a donor mismatched HLA antigen.

### 2.7. Statistical Analysis

Statistical analysis was performed using Fishers *T*-test and/or ANOVA. Allograft survival was analyzed using Kaplan Meier curves and the log-rank test.

## 3. Results and Discussion

IgG subclass analysis and how it is determined is a critical issue with regard to the interpretation of complement fixing and noncomplement fixing antibodies. We will endeavor herein to explicate the best way to determine the IgG subclasses of HLA antibodies. In the literature [12–15] comparative results for the IgG1 and IgG2 subclasses as correlate to outcomes should be viewed in the context of cross-reactivity. Therefore, in our results we present methodology for IgG subclass analysis.

Our data represent a unique group of patients with an average of greater than seven years of renal allograft function at the time of biopsy and DSA determination. Therefore it should be interpreted in this context.

**Figure 1:**
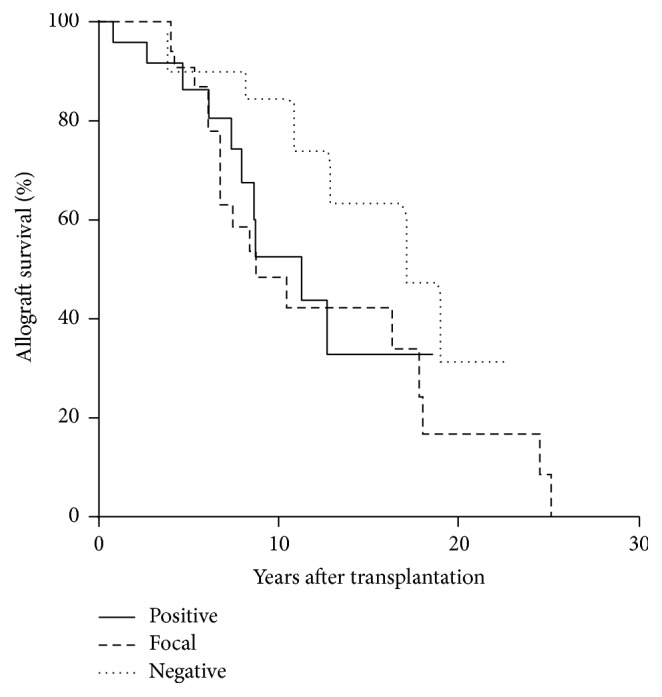
Comparison of renal allograft survival among study patients. The three cohorts of patients are separated according to the results of their with renal biopsies: diffusely positive C4d immunostaining (Positive), focally positive C4d immunostaining (Focal), and negative C4d immunostaining (Negative). The log-rank test result for the Kaplan Meier curves is 0.163.

The demographics of the studied patients are depicted in Table 1. There is a significant correlation to graft loss of the C4d+ and focally positive transplant recipients compared to the C4d− patients when analyzed by ANOVA (Table 1), but comparison of allograft survival among the groups did not reach significance using Kaplan Meier curves and the log-rank test (Figure 1). As noted, the average time of biopsy was greater than seven years after transplant, and some of these patients had incidences of rejection prior to inclusion in this study.

**Figure 2:**
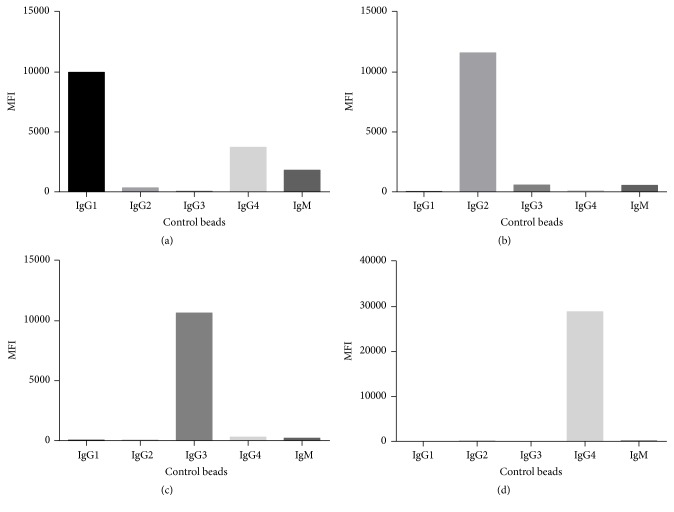
PE-conjugated secondary antibodies for IgG subclass detection. The control beads were incubated with negative control serum, washed, and then detected with subclass-specific antibodies ((a): IgG1-PE; (b): IgG2-PE; (c): IgG3-PE; (d): IgG4-PE).

**Figure 3:**
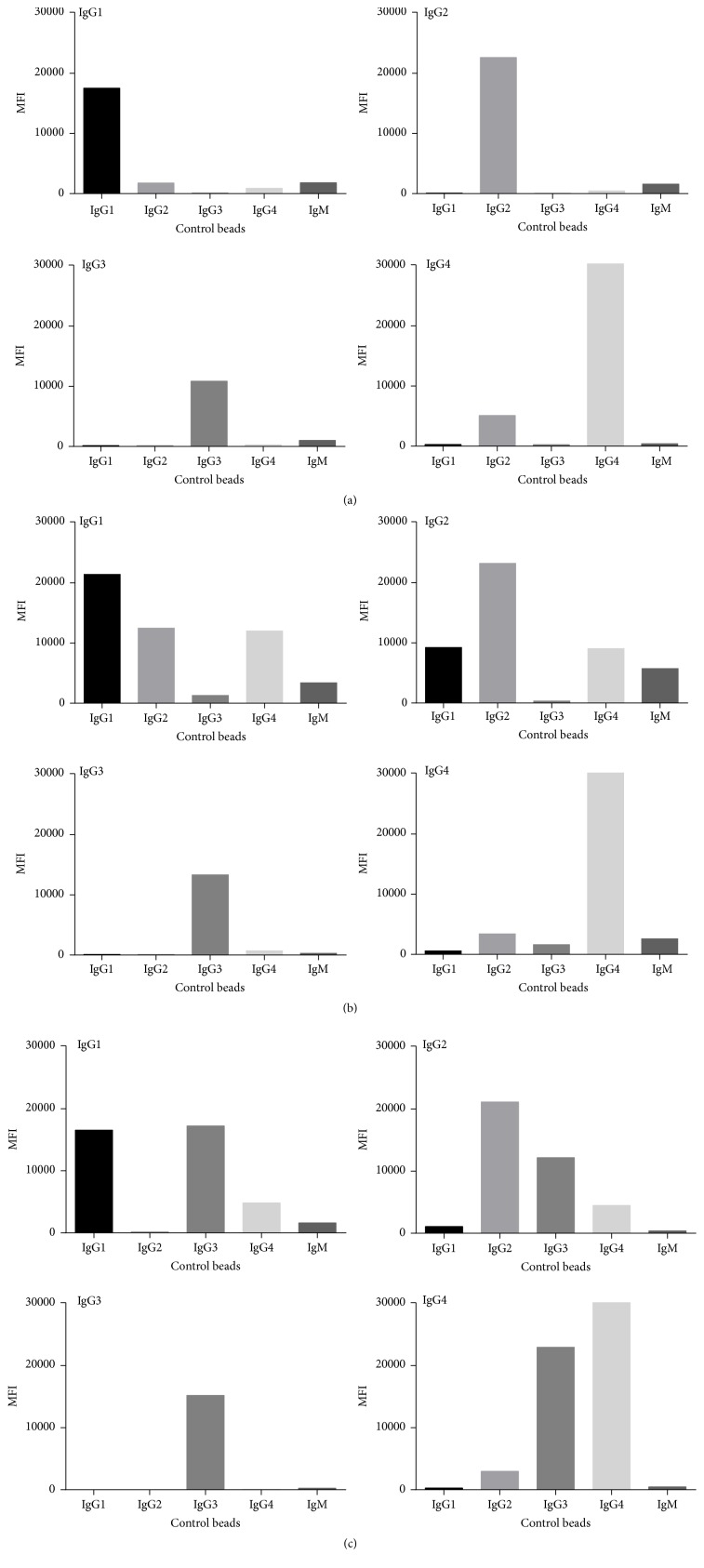
Examples of serum-dependent cross-reactivity of IgG subclass detection on control beads. The control beads were incubated with different patient sera, washed, and then detected with subclass-specific secondary antibodies ((a): patient serum 1; (b): patient serum 2; (c): patient serum 3).

The murine antibody clones that were utilized in the subclass experiments at concentrations selected to minimize cross-reactivity are shown in Figure 2 and Table 2. IgG1 had significant binding to the IgG4 control bead at the concentration at 1 *μ*g. These murine antibody clones were chosen from the available clones binding to the subclasses and represented the least cross-reactivity seen. The murine monoclonal subclass clones were the same as used in most of the subclass papers (Table 3) with the notable exception of IgG4 HP6023, which showed a strong MFI and was monospecific for IgG4. The concentration of IgG1 used by most of the investigators was different than used by us, and it is worth observing that individual lots of HP6001 can be quite different in cross-reactivity. Hence, it is problematic when switching lots, and IgG1 could give false reading for IgG4. In fact, Figure 3 shows the hypervariability of the murine monoclonal antibodies to IgG subclasses with three different sera on the control beads. As examples, Serum 1 shows results similar to that found in the PBS. Sera 2 and 3 show the extreme variation one finds with background in different sera. IgG3 and IgG4 showed the least serum-dependent differences. We point to these results as a caution in the interpretation of not only our results but those given by others, particularly for IgG1 and IgG2 and their propensity for cross-reactive results. We interpreted different serum binding results (Table 2) as nonspecific binding causing spurious results in the direct binding assay. However, in preliminary results when we used the indirect or “sandwich” assay at least some of the cross-reactivity could be eliminated (Table 4). We attribute the better results with the sandwich assay to the increased washing and secondary antibody binding, which we postulate caused elimination of lower affinity nonspecific adsorbed serum proteins causing cross-reactivity. Although our results below are based on the direct assay, we propose that the indirect assay provides better discrimination of IgG subclasses.

Table 5 shows a comparison of the cumulative DSA MFI in the patients, stratified by C4d status. In the C4d+ patients, all had DSA, and the average cumulative MFI was 12,353, as compared to the focally positive group average, which was 4,771 MFI. The predominant HLA for C4d+ patients was HLA Class II and also for the focally positive patients. However, in the focally positive group, nearly all of the DSA specificities were against HLA Class II and almost none against HLA Class I. The C4d− group had very low average DSA. One could argue that C4d− groups were potentially subclinical rejection, but it was clear from our results that in order to see C4d deposition on the PTC an average DSA of greater than 10,000 MFI was needed.

A more in depth analysis of DSA and C4d groups is presented in Table 6. All of the C4d positive groups were significantly positive when compared to the C4d−, and C4d+ DSA was significant when compared to the DSA in the focally positive group. This indicated that C4d reactivity was a function of the DSA at a certain threshold level. DSA used alone would predict a C4d+ result with a 95% confidence limit range between 6,436 and 16,343 MFI. C4d+ compared to focally positive C4d was significantly different, indicating that the DSA associated with C4d+ biopsies had significantly higher MFI. In addition, when we compared HLA Class I to HLA Class II in the focally positive group there was a significant difference, since the average HLA Class I DSA associated with the focally positive recipients were below 500 MFI. However, when HLA Class II DSA were compared between C4d+ and focally positive groups, there was no significant difference. Therefore, we saw that focally positive C4d and C4d+ were both associated with HLA Class II DSA but only C4d+ had a significant correlation to DSA in HLA Class I. Even though the focally positive C4d group tended had positive HLA Class II, it was only just significant compared to the C4d− with a broad 95% confidence limit range (351–7,716 MFI).

Table 7 shows standard DSA specificities, IgG subclass, and C1q all listed with MFI for the C4d+ patients. These results reiterate the specific antibodies that we saw in the analysis and the specific correlation of the results where there appears to be a threshold DSA for the C1q positive reaction. Also, one should note that the IgG subclass analysis seems to give more complete information regarding the complement fixing antibodies and IgG class switching. All of the patients had standard DSA, whereas seven patients had no C1q DSA, and this observation carries over into the total numbers of DSA for both HLA Class I and II. That is, fifteen patients had standard DSA to both Class I and Class II and only one class of C1q DSA. The threshold of DSA before seeing the C1q+ reactivity was ~4 DSA at 12,485 MFI and the C1q+ averaged 16,729 MFI with an average of only a single DSA (Table 8).

When we looked at the C4d+ patients outcomes there was no significant correlation with C1q or IgG subclass results (Table 9). There seemed to be a trend toward increased graft loss and thus possibly severity of rejection in the patients with C1q+ DSA. The profile of rejection and loss was in general a mixed, nonsignificant correlation. Looking at the pattern of C1q and IgG subclass DSA among the C4d+ patients (Table 10), we saw that all patients with complement fixing IgG1 subclass had DSA and eleven had IgG4 subclass. Also, 19 of 23 patients who were C4d+/DSA+ had complement fixing subclass DSA, as compared to 16 of 23 with C1q+ DSA. In this analysis, IgG1 subclass provided the best indication of the C4d+ reactivity, but was not a significant predictor of graft loss (Table 9). IgG4 subclass seemed to have little to do with the outcome and the complement fixation; that is, there was no blocking of the C4d+ complement fixation. An interesting result was the absence of IgG1 at the time of biopsy in four patients with significant dysfunction (average sCr of 4.4 mg/dL). Although only one of the four lost the transplant, it would be expected that there would be complement fixing antibodies. However, as noted above, the IgG1 assay could give quite variable results.

The absence of IgG3 subclass for the most part was likely a function of our longer term transplant patients that were analyzed. Also, patients had a large variation with regard to the cross-reactive of the IgG subclasses owing to the unknown variations intrinsically associated with the sera (Figure 2).

## 4. Conclusions

Many articles have been written regarding de novo DSA early after transplant [19–24]. The incidence of these de novo DSA ranged from 10 to 30% and the studies included 1–5 year follow-ups. In our study, a group of 73 patients were biopsied for cause with an average of greater than seven years after transplant, and the incidence of DSA was 41%. It is reasonable to suspect that patients biopsied for cause would have a higher incidence of DSA than patients without cause for biopsy. One should note that this is much less than has been reported by investigators using histology C4d criteria [11], but these patients likely exhibited accelerated acute rejection. However, 41% of patients with HLA DSA biopsied for cause can stand alone as a unique observation for our groups of patients biopsied after an average of seven years after transplant. There is a paucity in the literature of correlates between C4d+ and DSA MFI ranges but a few references show that immediately after transplant if DSA increase sharply or are greater than 9,500 MFI outcomes are significantly worse [20, 22]. A recent article reported on the diagnostic contribution of various assays to the diagnosis of silent AMR in renal transplant recipients [25]. This prospective study included patients who were determined to be DSA positive by protocol screening and underwent renal allograft biopsy, somewhat distinct from our patients who exhibited allograft dysfunction and were biopsied and tested for cause. The assays analyzed included the standard SAB assay, the C1q SAB assay, and a C3d SAB assay. The authors concluded that higher MFI DSA were associated with higher risk for rejection or allograft loss, but that the addition of complement fixing assays did not provide an enhanced diagnostic benefit. These results are concordant with our own findings, despite the differences in patient populations and utilized assays.

Our data show that the DSA in C4d+ patients was greater than 12,000 MFI, composed of both HLA Class I and II, and was significantly associated with C4d+ AMR. Also, focally positive patients had predominantly HLA Class II with an average of HLA Class I less than the 500 MFI negative cutoff (a consequence of the number of patients with no Class I DSA at all). Our observations showed that when C4d was focally positive there was dysfunction significantly associated only with HLA Class II. Others have shown that mismatching HLA Class II resulted in HLA-DQ DSA antibodies [26]. Certainly there may be occurrences where the DSA is lower but significant ACR glomerulopathy is present. However, for our C4d− group, the MFI range averaged just slightly above our negative cutoff of 500 MFI. Part and parcel of the C4d+ deposition on the PTC is the DSA detected in the serum but when one looks for antibodies on the PTC they are not present [27]. This may be a capping and stripping occurrence [28]. However, the important point may be that serum DSA is more stable and indicative of glomerulopathy and therefore suggests more attention be paid to DSA occurrence versus C4d. The question remains as to the quantity and the quality of the DSA present. In our study we have endeavored to quantify the DSA and also measure the complement activity of the antibodies. The measure of the complement inhibitory factors (CD55, CD45, and CD35) has yet to quantified but CD59 has been found on the PTC, thus inhibiting complement activity [29]. Again, this suggests that not just C4d but the quantity and quality of DSA are in general overarching factors in glomerulopathy.

Complement has a multitude of functions which include death of target cell or organism, proinflammatory effects, histamine release, phagocytosis, and chemotaxis [30, 31]. Therefore, looking for the activation of the complement pathway, assays have been developed that show activation of C1q as the initiation target which occurs when an antibody of the complement fixing class (IgM, IgG 1, 3) binds to the target HLA epitopes. The C1q assay should give a good concordance for with presence of damaging antibodies. Several authors have shown the effect C1q binding assay as a positive predictor of graft loss in de novo antibody patients, in both renal and heart transplants [32, 33]. However, there is a DSA threshold below which the C1q assay is negative, suggesting the quantity of the DSA antibody gives rise to the C1q+ result and the DSA MFI has the best predictive value for the C1q test. In our experience, C1q was negative in 30% of the DSA and C4d positive patients. Conversely, when complement fixing antibody subclasses were measured directly only 18% were negative. Furthermore, when C1q was positive, DSA was greater than 12,000 MFI and always appeared in the presence of C4d+ DSA+ plus complement fixing subclass antibodies. Among our patients who tested negative for C1q DSA, there were three patients who had complement fixing IgG subclass antibodies in the presence of DSA and C4d+ results.

IgG subclasses as mentioned above were a better correlate for complement fixation compared to C1q in the C4d+ DSA patients. We feel that IgG subclasses give a more complete picture of the complement fixing antibodies present. Indeed, strong complement fixing IgG3 along with DSA had the highest risk for graft loss in liver transplant recipients [34]. C1q+ correlated with high risk of graft loss but seemed to add little to the risk associated with IgG3+, DSA+. In our current study, there was a paucity of IgG3+ recipients, possibly owing to the average posttransplant biopsy times. That is, IGg3 is the first complement fixing antibody in class switching from IgM and may not be present except in acute, initial AMR response which is in contrast with our greater than seven years posttransplant, biopsied patient population. Interestingly, in the 82% of patients IgG1+ C4d+, eleven patients had antibodies of the IgG4 subclass, which is one of the last antibodies to class switch. IgG4 has been often described as a blocking antibody, because it does not fix complement. IgG4 has been found in some patients to be present solely and with salutary effect for transplantation [16]. However, in this study we could not see any association between the presence of IgG4 DSA and lower sCr or better graft survival. One study suggests that IgG4 has a detrimental effect on graft outcomes [35]. We suggest because class switching is governed by time and T regulatory cells [36], when IgG4 occurs in these long term biopsied patients it may be associated with accommodation and T regulatory cell but concomitant presence of IgG1 negates these beneficial effects. In a recent case [37], we found a patient C4d− DSA+ C1q+ IgG1+ and IgG4+ (22,000 MFI) and a sCr < 0.9 mg/dL. The patient was treated for rejection with IVIg, but this therapy was discontinued because of infusion reactions. In this case, it is possible that the IgG4 antibody was blocking the complement fixation in vivo. Indeed we could show CDC blocking by this patient's serum in vitro in preliminary experiments using human serum as a complement source (unpublished data). Thus, if an IgG4 antibody is present in high enough concentration we expect that the cross-linking of two immunoglobulin molecules is blocked and complement is not activated. Such a mechanism may not occur in the C1q bead assay because of the quantity and proximity of the HLA bound to the beads.

The presence of IgG4 in transplant recipients may be most analogous to the phenomenon observed in subcutaneous allergic desensitization. Allergic desensitization occurs after weekly subcutaneous allergen injection and successful results show IgG4 specific allergen antibodies. The class switching is governed by T regulatory cells secreting IL-10 and TGF-*β* predominantly [36]. Thus the allograft may in many cases, when DSA is present, eventually cause the switch to IgG4 and a T regulatory phase. However, it may be too late for the allograft since the ongoing presence of complement fixing antibodies typically precedes IgG4. We have proposed using the allergic desensitization model in pretransplant patients with high cPRA using HLA proteins specific to the detected antibodies [16].

Lastly, IgG1 and IgG2 subclasses have a very high variability regarding their cross-reactivity when detected by the available murine antibodies (Table 4). Although in this study we used the direct assay for subclass identification, we have seen less cross-reactivity using an indirect sandwich assay. We postulate that the extra washing and additional antibody strip off the lower affinity cross reacting antibodies. We feel utilization of a standard indirect assay technique can bring about a better correlation to the complement fixing antibodies and aid in our understanding of the presence or absence of subclass antibodies using standardized kits to ascertain IgG subclass values.

## Figures and Tables

**Table 1:** Patient demographics.

	C4d diffusely positive (*N* = 23)	C4d focally positive (*N* = 25)	C4d negative (*N* = 25)	*P* value
Age				
At biopsy [yr.]	44.5 ± 12.7	45.7 ± 9.9	49.5 ± 13.7	0.343
At transplantation [yr.]	39.0 ± 10.9	37.1 ± 12.1	42.1 ± 13.3	0.356
Female sex [%]	7 (30.4)	8 (32.0)	8 (32.0)	0.938
Race or ethnic group [%]				0.104
White Hispanic	15 (65.2)	11 (44.0)	16 (64.0)	
Asian	2 (8.7)	4 (16.0)	2 (8.0)	
Black non-Hispanic	4 (17.4)	1 (4.0)	1 (4.0)	
Mixed	0 (0.0)	4 (16.0)	0 (0.0)	
White non-Hispanic	3 (13.0)	2 (8.0)	5 (20.0)	
White unknown	0 (0.0)	1 (4.0)	0 (0.0)	
Native American	0 (0.0)	1 (4.0)	0 (0.0)	
Previous Transplantation [%]	7 (30.4)	2 (8.0)	4 (16.0)	0.168
Simultaneous kidney pancreas [%]	1 (4.3)	3 (12.0)	1 (4.0)	0.423
Deceased donor [%]	15 (65.2)	20 (80.0)	19 (76.0)	0.211
Cause of ESRD [%]				0.868
Diabetes	4 (17.4)	4 (16.0)	7 (28.0)	
Hypertension	8 (34.8)	7 (28.0)	7 (28.0)	
Miscellaneous	9 (39.1)	10 (40.0)	9 (36.0)	
Unknown	3 (13.0)	3 (12.0)	1 (4.0)	
Allograft year at Bx [yr.]	5.4 ± 5.0	8.7 ± 6.2	7.4 ± 5.6	0.138
Allograft loss [%]	9 (39.1)	16 (64.0)	7 (28.0)	0.023
Graft year at loss [yr.]	7.8 ± 3.1	11.1 ± 7.0	10.9 ± 6.0	0.61

**Table 2:** PE conjugated secondary antibodies for IgG subclass detection.

Secondary Ab	MFI [relative%]				
	IgG1 bead	IgG2 bead	IgG3 bead	IgG4 bead	IgM bead
IgG1-PE 1 *µ*g/mL HP6001	9989 (100)	341 (3)	27 (0)	3733 (37)	1814 (18)
IgG2-PE 5 *µ*g/mL HP6014	31 (0)	11621 (100)	576 (5)	117 (1)	556 (5)
IgG3-PE 5 *µ*g/mL HP6050	19 (0)	21 (0)	10666 (100)	307 (3)	215 (2)
IgG4-PE 1 *µ*g/mL HP6023	5 (0)	113 (0)	12 (0)	28872 (100)	50 (0)

**Table 3:** Reported IgG subclass antibodies used for DSA detection.

		IgG1	IgG2	IgG3	IgG4	Control
Cicciarelli et al. (2013) [16]	Clones	HP6001	HP6014	HP6050	HP6023	Control bead
	Concentration	1 *μ*g/mL	5 *μ*g/mL	5 *μ*g/mL	1 *μ*g/mL	
	MFI					

Lefaucheur et al. (2016) [17]	Clones	HP6001	31-7-4	HP6050	HP6025	Negative sera
	Concentration	5 *μ*g/mL	5 *μ*g/mL	20 *μ*g/mL	20 *μ*g/mL	
	MFI	Mean Pan-IgG 1784				

Hönger et al. (2011) [14]	Clones	HP6001	31-7-4	HP6050	HP6025	Negative sera
	Concentration	1.3 *μ*g/mL	1.3 *μ*g/mL	10.6 *μ*g/mL	0.68 *μ*g/mL	
	MFI	Median, 1524; range, 1083–3584, ratio above the cutoff (i.e., ratio = MFI IgG subclass divided by MFI cutoff)				

Kaneku et al. (2012) [15]	Clones	4E3	HP6002	HP6050	HP6025	Control bead
	Concentration	?	?	?	?	
	MFI	Median IgG subclass MFI in chronic rejection patients with post-OLT DSA (71%) was 5596				

Khovanova et al. (2015) [13] Lowe et al. (2013) [18]	Clones	4E3	31-7-4	HP6050	HP6025	Control bead
	Concentration	250 *μ*g/mL	250 *μ*g/mL	250 *μ*g/mL	250 *μ*g/mL	
	MFI	Median MFI of IgG ~1000				

Arnold et al. (2014) [12]	Clones	HP6001	31-7-4	HP6050	HP6025	Isotype control
	Concentration	?	?	?	?	
	MFI	?	?	?	?	

**Table 4:** Comparison of direct and sandwich IgG subclass assays.

		IgG1 beads	IgG2 beads	IgG3 beads	IgG4 beads	IgM beads
Sandwich assay	IgG1 HP6001	100%	14%	41%	15%	2%
	IgG2 HP6002	8%	100%	27%	25%	2%
	IgG3 HP6047	1%	0%	100%	4%	0%
	IgG4 HP6023	1%	1%	5%	100%	0%

Direct assay	IgG1 4E3	100%	228%	260%	5%	1%
	IgG1 HP6001	100%	2%	88%	3%	1%
	IgG2 HP6002	5%	100%	105%	126%	6%
	IgG2 31-7-4	14%	100%	245%	185%	2%
	IgG3 HP6050	0%	0%	100%	1%	0%
	IgG4 HP6023	0%	0%	2%	100%	0%

**Table 5:** Reactivity of monoclonal IgG subclass detection antibodies against control microbeads.

	C4d+ mean MFI	C4d focally positive mean MFI	C4d− mean MFI
Total	12,353	4,771	963
Class I	5,487	443	442
Class II	7,373	4,317	520

MFI cutoff for DSA positivity set at 500.

**Table 6:** In depth analysis of DSA and C4d+, C4d focally positive, and C4d− patient groups.

	*P* value	95% confidence interval MFI
C4d+ DSA MFI versus C4d− DSA MFI	<0.0001	6,436–16,343
Class I C4d+ DSA MFI versus Class I C4d− DSA MFI	<0.0005	2,473–7,616
Class II C4d+ DSA MFI versus Class II C4d− DSA MFI	<0.0011	3,053–10,652
C4d+ DSA MFI versus C4d focally positive DSA MFI	<0.012	1,718–13,443
Class I C4d+ DSA MFI versus Class I C4d focally positive DSA MFI	<0.0005	2,455–7,634
Class II C4d+ DSA MFI versus Class II C4d focally positive DSA MFI	<0.2	NS
Class I C4d focally positive DSA MFI versus Class I C4d− DSA MFI	<0.4	NS
Class II C4d focally positive DSA MFI versus Class II C4d− DSA MFI	<0.0431	351–7,716

**Table 7:** Pan-DSA specificities, IgG subclass, and C1q in C4d+ patients.

Patient no.	PAN-IgG				IgG1				IgG2				IgG3				IgG4				C1q	
	Class I		Class II		Class I		Class II		Class I		Class II		Class I		Class II		Class I		Class II		Class I	Class II
1	B7	1608																			0	0

2	Cw7	16888			Cw7	14869			Cw7	5271							Cw7	752			20236	0
	B44	2233																				
	A68	1040																				

3	Cw7	9642	DQ6	4396	Cw7	4509	DQ6	1190													11331	0
	A30	6939			A30	1254																
	B8	5643																				

4	B7	16700			B7	4733															1604	0
	Cw15	6627																				
	Cw10	5268			Cw10	1420																
	A31	1988																				

5	Cw1	1349	DP2	11354			DP2	3714											DP2	2462	0	0
	Cw10	1342	DR4	2718															DR4	5517		
	B55	1035	DQ9	2155																		
			DP5	1603																		
			DQ4	1366																		

6	A11	1371	DQ5	4682			DQ5	3376													0	0
			DR51	2765																		
			DR16	1351																		

7	A2	999	DQ2	3350			DQ2	2884							DQ2	714					0	5892
	Cw4	1000	DR52	926	Cw4	1071																

8	B35	4059	DQ4	6038			DQ4	2747			DQ4	6612							DQ4	5397	0	16072
	B61	843	DR53	673																		

9	A2	2647	DR52	1099	A2	1099															0	0

10	Cw4	3039			Cw4	4541															0	0
	A33	1028																				
	A68	924																				
	B53	879																				

11	A31	5220	DR7	11314	A31	2195															20551	0
	B65	4383	DQ2	5223	B65	2129	DQ2	701			DQ2	1012										
	B64	2680			B64	867																
	B51	2458																				

12	A68	13440	DQ8	14106	A68	6021	DQ8	4727									A68	779	DQ8	17214	509	50289
	B49	6052	DQ9	13986	B49	1238	DQ9	5506			DQ9	982							DQ9	18600		
	A1	2023	DQ7	11822			DQ7	4283			DQ7	1978							DQ7	17779		
			DR4	1800																		

13	A31	13834	DR8	3593	A31	4506	DQ4	2386													24668	0
	A30	6813	DQ4	3289	A30	2171	DR8	1270														

14			DQ7	12435			DQ7	4488			DQ7	4352							DQ7	7343	0	28347

15	A24	4141	DQ7	3611	A24	9132	DQ7	3503									A24	7291	DQ7	4555	0	7411
	Cw4	1881	DR52	1271	Cw4	8717											Cw4	4366				

16	A31	11549			A31	3902							A31	2242			A31	7998			25084	0

17	A32	1193	DR1	1732																	0	0
			DR51	1127																		

18																					0	28441

19	A2	4105	DQ7	8907	A2	2112	DQ7	4052	A2	4099	DQ7	1702	A2	4181	DQ7	843	A2	19117	DQ7	1782	0	26224
	A29	1442			A29	1585																
	Cw7	1078			Cw7	717																

20			DQ5	8804			DQ5	4457											DQ5	2988	0	28688
			DQ4	5672			DQ4	2743														
			DR16	925																		

21			DQ5	9454																	0	1836
			DR1	1883																		
			DR4	1712																		

23	A31	3505																			0	0

24	B62	894	DR53	8265			DR53	954													0	16627
			DR7	2371																		
			DR4	1771															DR4	1214		

**Table 8:** DSA, IgG subclasses, and C1q detection in C4d+ patients.

Assay	Type of DSA detected [# of patients]			
	No DSA	Class I only	Class II only	Class I+II
Standard	0	5	3	15
C1q	7	6	9	1

Assay	Mean total DSA MFI			Average # of DSA
		Class II only	Class I+II	

Standard	7,046	8,320	12,485	4
C1q	14,460	17,864	16,729	1

**Table 9:** Analysis of C1q, IgG subclass, and Cd4d+ DSA.

	C1q− (*N* = 7)	C1q+ (*N* = 16)	IgG1− (*N* = 4)	IgG1+ (*N* = 19)	IgG1+ IgG4− (*N* = 8)	IgG1+ IgG4+ (*N* = 11)
Mean sCr (at biopsy)	3.5 mg/dL	2.9 mg/dL	4.4 mg/dL	2.8 mg/dL	2.7 mg/dL	2.9 mg/dL
Mean sCr (most recent)	2.2 mg/dL	1.9 mg/dL	3.4 mg/dL	1.9 mg/dL	1.8 mg/dL	1.9 mg/dL
Graft loss (*N* = 5)	1 (14%)	4 (25%)	1 (25%)	4 (21%)	1 (13%)	3 (27%)

Values did not reach statistical significance.

**Table 10:** DSA detected by C1q and IgG subclass.

Patient	C1q	IgG1	IgG2	IgG3	IgG4
1	**+**	**+**	**+**		**+**
2		**+**			
3		**+**			
4		**+**			**+**
5	**+**	**+**			**+**
6	**+**	**+**			
7	**+**	**+**		**+**	
8	**+**	**+**	**+**		**+**
9	**+**	**+**			
10		**+**			
11	**+**	**+**	**+**		
12	**+**	**+**			
13	**+**	**+**	**+**		**+**
14	**+**	**+**			**+**
15	**+**	**+**		**+**	**+**
16					
17	+	+	+		**+**
18	**+**	**+**	**+**	**+**	**+**
19	**+**	**+**			**+**
20	**+**				
21	**+**	**+**	**+**		**+**
22					
23	**+**	**+**			**+**
